# Using Kirkpatrick’s model to measure the effect of a new teaching and learning methods workshop for health care staff

**DOI:** 10.1186/s13104-019-4421-y

**Published:** 2019-07-10

**Authors:** Mohammad Reza Heydari, Fatemeh Taghva, Mitra Amini, Somayeh Delavari

**Affiliations:** 1 HIV/AIDS Research Center, Institute of Health, Shiraz University of Medical Sciences, Shiraz, Iran; 2 Shohaday-Enghelab Health Center, Shiraz University of Medical Sciences, Shiraz, Iran; 3 Clinical Education Research Center, Shiraz University of Medical Sciences, Shiraz, Iran; 4 Center for Educational Research in Medical Sciences (CERMS), Department of Medical Education, School of Medicine, Iran University of Medical Sciences, Tehran, Iran

**Keywords:** Kirkpatrick’s model, Teaching methods, Workshop

## Abstract

**Objectives:**

This study is designed to evaluate the effect of a workshop about new teaching and learning methods on the response, knowledge, and behavior of healthcare staff working a large city healthcare center.

**Results:**

Kirkpatrick’s program evaluation model showed that the workshop on new teaching and learning methods significantly improved the healthcare staff’s satisfaction about the teaching environment of workshops, their knowledge about new teaching and learning methods and their behavior in performing workshops for teaching people. It is recommended that this teaching and learning methods workshop should be considered in educational programs for healthcare staff.

*Trial registration* Trial registration number: IRCT20180619040150N1 approved by Iranian Registry of Clinical Trials at 2018-07-27

**Electronic supplementary material:**

The online version of this article (10.1186/s13104-019-4421-y) contains supplementary material, which is available to authorized users.

## Introduction

Training is a useful investment and is one of the most important factors in human resource development. When done well, it can improve employee satisfaction, intended outcomes, and economical efficiency [1, 2]. Many factors can affect whether training programs achieve their desired outcomes [3, 4]. One of the most critical parts of implementing training programs is accurately assessing their impact; to assess programs requires using a suitable method [5]. Here, in addition to measuring learners’ satisfaction with self-assessment [6], meaningful assessment must measure input (learners and teachers), training process (educational programs, assessment methods, and facilities), and output (behavior of participants) [7]. One of the methods used to assess educational programs is Kirkpatrick’s model. The characteristics of this model include the simplicity of the process, measurement of a limited number of variables, ease of evaluation criteria, and lack of need to collect the basic data and learners’ previous performance, and independence of individual and environmental variables. This type of assessment is an appropriate model for evaluating educational programs [8]. Although all models have some deficiencies, according to the evaluation, this model has a suitable and acceptable performance for assessing the educational programs [9].

Kirkpatrick’s model assesses the effectiveness of training programs at four levels: (1) response of the trainee to the training experience (including training experience); (2) the learner’s learning outcomes and increases in knowledge, skill, and attitude towards the attendance experience (how much attendees learned the content after training). This level usually measured through using a pretest and posttest; (3) the students’ change in behavior and improvement (whether the learning transferred into practice in the workplace); and (4) results (the ultimate impact of training) [8, 10–12].

Since healthcare staff holds various courses for the people across all sections and levels, it is necessary for these staff to become familiar with new teaching and learning methods. Thus, this study used Kirkpatrick’s program evaluation model to evaluate the effect of workshops about new teaching and learning methods on the healthcare staff’s satisfaction, learning and behavior.

## Main text

### Method

The present study involved a pretest, educational intervention, and posttest.

$${\text{O}}_{ 1} \quad {\text{X}}\quad {\text{O}}_{ 2}$$

O_1,2_ is the Observation of the dependent variable (pretest and posttest), X is the Exposure to the educational intervention, the independent variable (workshop).

#### Participants

This study was a quasi-experimental study conducted on the healthcare staff working in the Shiraz healthcare center.

#### Educational intervention

A workshop entitled “New Teaching and Learning Methods” was held over 2 days (10 h total) for the healthcare staff. In this workshop, participants learned to teach and learn in small groups, role-playing, brainstorming, question and answer, interactive lecture, and team-based learning. The teachers of these workshops were medical education experts working at the Education Development Center of Shiraz University of Medical Sciences. The content of workshops was about how to deliver an interactive lecture, small group teaching and learning, classroom management, time management, group dynamic and etc.

#### Assessing workshop and data gathering tools

We evaluated the educational effect of this workshop according to the first three levels of Kirkpatrick’s model using two questionnaires and one checklist.

The first step of the Kirkpatrick program evaluation model is related to the participants’ reactions [8]. Thus, in this step, participants’ attitudes were evaluated regarding the effect of training on their educational ability via a researcher-administered questionnaire. Accordingly, the first level participants’ reaction toward the training course measured with a researcher-made questionnaire. The questionnaire had three categories including: instructor assessment, course content assessment and course support assessment that were scored based on a 5-point Likert scale from excellent to very weak. At the end of the questionnaire there were two questions about overall quality of workshops and overall satisfaction of participants. The validity of the questionnaire was determined by medical education experts and the reliability was confirmed after a pilot study (r = 0.83). A sample of questionnaire is in Additional file 1: Appendix S1.

In the second level of the Kirkpatrick program evaluation model, the effect of the program was evaluated on participants’ learning using pre- and post-test [11]. Accordingly, this questionnaire used before and after workshop which had ten questions including themes as educational goals, entrance exam, teaching time, and trainees’ preparation, the use of new teaching and learning methods, use of learning tools, teaching environment, the number of participants, teacher assessment, and teacher characteristics. The score of each item in the questionnaire was 1, and the total score of this questionnaire was 10. The validity of the questionnaire determined by medical education experts and the reliability determined after a pilot study (r = 0.81).

In the third level Kirkpatrick program evaluation model, we assessed whether the learning was transformed into practice in the workplace [8, 10, 11] by using an observational checklist. This checklist contains some parts about using new teaching and learning methods in the educational sections held by the healthcare staff. Before intervention and 2 months after intervention, two expert observers attended at all of the healthcare staff educational sections and assessed the sections based on this checklist. Every healthcare worker was conscious about the observers and for lowering the effect of the under-observation stress, the healthcare staff participated behavior in their own educational sessions assessed after participating in the workshop of new teaching methods and compared it with the results of one their educational sections before the educational intervention. Besides for lowering the effect of under observation stress, the observers attended at five educational sessions before and at five educational sessions after intervention and completed the checklist after at least half of the sessions to be sure about lowering the stress of healthcare workers.

#### Statistical analysis

The data were analyzed through SPSS 21, using paired t-test.

### Results

This study was an interventional study conducted on 48 staff working in the Shiraz healthcare center. Among them, 12 (25%) were male, and 36 (75%) were female. The mean age of the participants was 40 ± 12 years and their mean work experience was 18 ± 8 years.

The results of the first level Kirkpatrick evaluation indicated that 30 subjects (62.5%) who participated in the new teaching and learning methods workshop declared that the quality of this workshop was excellent. Thirty-five participants (72.9%) were completely satisfied with the way of conducting workshops. In all other domains of the questionnaire more than 50% of participants declared that the quality was excellent.

The results of the second level Kirkpatrick’s evaluation about the participants learning revealed that there was a significant difference between the total scores, preparation of trainees, and use of new teaching and learning methods before and after the intervention (p < 0.05), but there were not any significant differences between other factors (Table 1).

**Table 1:** The scores of experts participating in the workshop before and after the intervention (second level)

Evaluation of the second level Kirkpatrick model	Mean score (SD)		p-value
	Before intervention	After intervention	
How to define educational goals	0.22 (0.15)	0.23 (0.2)	0.822
How to use pre tests	0.16 (0.05)	0.23 (0.2)	0.160
How to set class time	0.37 (0.21)	0.38 (0.21)	0.743
How to prepare trainees	0.26 (0.25)	0.4 (0.2)	0.003
How to use new teaching and learning methods	0.4 (0.04)	17 (0.0.9)	0.003
How to use learning assistant tools	0.73 (0.27)	0.78 (0.29)	0.352
How to design learning environment	0.61 (0.51)	0.5 (0.08)	0.355
How to set teaching based on the number of participants	0.22 (0.2)	0.25 (0.15)	0.660
How to use appropriate evaluation methods	0.62 (0.35)	0.7 (0.33)	0.309
How to be a good teacher	0.54 (0.09)	0.76 (0.37)	0.06
Total score	5.62 (1.58)	6.66 (1.25)	0.001

To measure the third level of Kirkpatrick’s program evaluation method, the researchers observed the participants’ behavior during the workshops performed by them 2 months after their training. According to the results, the behavior change across all of the dimensions was significant (p ≤ 0.05) except in using pretests, defining educational goals, time management, defining key points of lecturing, using role play method, and speech structure (Table 2).

**Table 2:** Evaluating workshop held by participants before and after intervention according to the items related to third level Kirkpatric’s evaluations

No.	Evaluation of the third level Kirkpatrick model	Number and percent of participants		p-value
		Before intervention	After intervention	
1	Using pre tests	30 (75%)	35 (78.5%)	0.180
2	Defining educational goals	17 (42.5%)	22 (55%)	0.179
3	Time management	33 (82.5%)	32 (80%)	0.977
4	Focusing on trainee during speech	26 (65%)	39 (97.5%)	0.001
5	Defining key points of lecturing	26 (65%)	31 (77.5%)	0.180
6	Preparation of trainees	36 (90%)	48 (100%)	0.001
7	Using role play method	22 (55%)	25 (62.5%)	0.629
8	Using group discussion	28 (70%)	36 (90%)	0.039
9	Using brainstorming technique	9 (22.5%)	20 (50%)	0.007
10	Speech structure	23 (57.5%)	30 (75%)	0.189
11	Using problem solving	19 (47.5%)	36 (90%)	0.001
12	Using appropriate questions	26 (65%)	37 (92.5%)	0.0013
13	Appropriate group dynamic	26 (65%)	35 (87.5%)	0.035
14	Using problem based learning	29 (72.5%)	36 (90%)	0.004
15	Using verbal and nonverbal communication skills	19 (47.5%)	36 (90%)	0.001
16	Sitting arrangement of teacher and trainees	19 (47.5%)	34 (85%)	0.001
17	The overall using of small group	22 (55%)	37 (92.5%)	0.001
18	Cooperative learning in small group	13 (32.5%)	31 (77.5%)	0.001

### Discussion

Each program evaluation model has strengths and weaknesses to measure training activities, but research has shown that the Kirkpatrick’s program evaluation model is more appropriate than other models [13, 14]. Accordingly, we used the Kirkpatrick’s model to evaluate the healthcare staff’s overall reaction to the workshop for new teaching and learning methods and its effects on their learning and behavior.

The results of the workshop evaluation in the reaction part (Level 1) showed that the participants were satisfied. A study conducted by Rabiee et al. on holding training courses for the staff working in Arak University of Medical Sciences suggested that half of the staff believed that the workshop was perfect while the other half thought that it was moderate or weak [15]. Other research conducted by Opoghi et al. to evaluate the librarians’ reaction toward a short-term training course using Kirkpatrick’s model revealed that there was a direct and positive relationship between the physical conditions of class and quality of learning [16].

Evaluation of the second level of Kirkpatrick’s model demonstrated a significant difference between the participants’ learning scores before and after the intervention. The overall results of this study suggested an increase in learning and satisfaction and changes in behavior scores. Dorri et al. also examined the effect of in-service training on cardiopulmonary resuscitation using Kirkpatrick’s model. They found it effective in increasing the participants’ learning and knowledge [17]. Poujahromi et al. conducted a study using Kirkpatrick’s model to evaluate a course on working with DC shock held for nurses working in Bushehr Hospital. The results were in line with this study. Another study performed by Anderson et al. in 2000 revealed educational success and organizational effectiveness in the use of this training method. They found a significant difference between educational assessment and educational achievement [18].

Mbagwu’s research in 2010, which was conducted on employees at the Federal University of Technology Library, showed that these training courses increased their capabilities, skills, and specific knowledge [19]. He emphasized that the training courses must be presented using a logical and appropriate method. Dorri’s research study dealt with the effectiveness of training courses using Kirkpatrick’s model. He observed that any changes in each of the four dimensions (reaction, learning, behavior, and results) were influential [17]. Abedini conducted a study to evaluate the effectiveness of in-service training courses from the perspective of employees working in Tehran Maskan Bank. He concluded that the participants assessed the behavioral changes at a reasonable level [20].

A brief review and overview of the process of investing in educational activities all over the world in recent years suggested that the amount of resources dedicated to this critical issue is growing compared to previous years. Nevertheless, some concerns still observed on the part of the officials and decision-makers about the lack of effectiveness of educational activities. The results of Farjad et al. indicated that although training courses can partially enhance skills and increase the scores of Kirkpatrick’s levels, a lack of attention to new and effective teaching and learning methods reduces their usefulness [21].

Integration of the health system and medical education in Iran prepare a unique environment that allows university educational experts to teach healthcare staff [22]. Good program evaluation models are needed to measure the effectiveness of these important educational activities.

Performing this kind of workshop familiarizes the healthcare staff with the organization’s new teaching and learning methods. Workshops can also be effective in motivating the staff. According to the results, holding workshops on new teaching and learning methods significantly improved the healthcare staff’s satisfaction and their function after the intervention.

Aside from curriculum and training delivery, results showed that the training about new teaching and learning methods is an essential factor in delivering effective teaching by healthcare staff. This staff is responsible for educating people in urban and suburban areas all over the country, so improving their teaching methods to deliver educational content effectively will lead to an increase in population health.

Also, health care staff help to promote knowledge, attitudes, and behaviors within the community on common diseases. The results of our study indicate that healthcare staff training can be used as an efficient and low-cost method for providing education to all of the community.

## Limitations

The limitations of the present study were the short duration of workshops and the small number of participants. Another limitation was that measuring the fourth level of the Kirkpatrick model was not possible.

## Additional file

**Additional file 1.** 1st level Kirkpatrick’s questionnaire.

## Data Availability

The datasets used and/or analyzed during the current study are available from the corresponding author on reasonable request.
