# Supplementary material for: Using Kirkpatrick’s model to measure the effect of a new teaching and learning methods workshop for health care staff
Source: BMC Res Notes. 2019 Jul 10;12:388. doi: 10.1186/s13104-019-4421-y (PMC6617554; doi:10.1186/s13104-019-4421-y)
Supplement: Supplementary file 1 — Additional file 1. 1st level Kirkpatrick’s questionnaire. [file 13104_2019_4421_MOESM1_ESM.docx]

Dear Colleague

Improving the quality of in-service training in the University requires your constructive and insightful comments and recommendations.

Please assist us in provision of effective training conditions by answering to the following questions accurately.

Training course title: Course Start Date: Course End Date:

Duration of the course:

**1^st^ level Kirkpatrick’s questionnaire**

| **Instructor Assessment** | **excellent** | **Very good** | **Intermediate** | **weak** | **Extremely weak** |
| --- | --- | --- | --- | --- | --- |
| Academic proficiency of the instructor |  |  |  |  |  |
| Lecturing method and the ability to transfer the concepts to learners |  |  |  |  |  |
| Ability of the instructor in class management |  |  |  |  |  |
| Use of active teaching methods and engaging the learners |  |  |  |  |  |
| Ability to respond to the ambiguities and questions of learners |  |  |  |  |  |
| Frequency of using practical examples during teaching |  |  |  |  |  |
| **Course content assessment** | **excellent** | **Very good** | **Intermediate** | **weak** | **Extremely weak** |
| Effectiveness of the contents of the course in increasing your knowledge |  |  |  |  |  |
| Relationship between training course and your organizational needs |  |  |  |  |  |
| Up-to-datedness of the contents of the course |  |  |  |  |  |
| Quality of teaching in the training course |  |  |  |  |  |
| **Course support assessment** | **excellent** | **Very good** | **Intermediate** | **weak** | **Extremely weak** |
| Your satisfaction with the duration of the course |  |  |  |  |  |
| Desirability of educational location and environment |  |  |  |  |  |
| Brightness and light in the classes |  |  |  |  |  |
| Ventilation and adequacy of cooling/heating system |  |  |  |  |  |
| Catering and reception |  |  |  |  |  |
| Treatment of authorities toward you |  |  |  |  |  |
| **Overall satisfaction** | **excellent** | **Very good** | **Intermediate** | **weak** | **Extremely weak** |
| Satisfaction of the quality of workshops |  |  |  |  |  |
| Satisfaction of the way of conducting workshops |  |  |  |  |  |

If you have any recommendations in addition to the above cases to increase the quality of the courses, please write them here.
